# Secukinumab-Aggravated Disseminated Discoid Lupus Erythematosus Misdiagnosed as Psoriasis

**DOI:** 10.7759/cureus.72698

**Published:** 2024-10-30

**Authors:** Binrong Ye, Dongmei Liu, Chunyan Tu, Shuai Yan, Yeqiang Liu

**Affiliations:** 1 Dermatology, Suzhou Traditional Chinese Medicine (TCM) Hospital Affiliated to Nanjing University of Chinese Medicine, Suzhou, CHN; 2 Dermatology, The Fifth Hospital of Jinhua City, Jinhua, CHN; 3 Gynecology, Suzhou Traditional Chinese Medicine (TCM) Hospital Affiliated to Nanjing University of Chinese Medicine, Suzhou, CHN; 4 Proctology, Suzhou Traditional Chinese Medicine (TCM) Hospital Affiliated to Nanjing University of Chinese Medicine, Suzhou, CHN; 5 Dermatology, Shanghai Skin Disease Hospital ,Tongji University, Shanghai, CHN

**Keywords:** biologics, discoid lupus erythematosus, lupus, psoriasis, secukinumab

## Abstract

Discoid lupus erythematosus is an autoimmune disease. When the disease manifests in multiple sites, it is classified as disseminated discoid lupus erythematosus. It is challenging for dermatologists to distinguish this condition from other erythematous diseases, and it is sometimes misdiagnosed as psoriasis. Dermoscopy and pathology biopsy are essential supplementary diagnostic tools. Furthermore, biologics utilized for psoriasis treatment may exacerbate the condition if incorrectly administered to patients with discoid lupus erythematosus. Herein, we present a case study in which disseminated discoid lupus erythematosus was misdiagnosed as psoriasis and worsened after the use of an interleukin-17 inhibitor known as secukinumab.

## Introduction

Discoid lupus erythematosus (DLE) is a form of cutaneous lupus. Erythematous plaques, distinct demarcations, adherent thick scales, and follicular plugging are characteristics of DLE. Its pathology is characterized by interfacial dermatitis with vacuolar degeneration of basal keratinocytes, hyperkeratosis with follicular plugging, and perifollicular infiltration. Superficial and deep dermal lymphocytic infiltration and interstitial mucin deposition are present. When lesions manifest in multiple sites throughout the body, the condition is referred to as disseminated DLE. Disseminated DLE presents with a variety of lesions that may be misidentified as other erythematous conditions, such as psoriasis, particularly when observed in the absence of other clinical information [1]. Auspitz's sign and dermoscopy are instrumental in differentiating between DLE and psoriasis and are indispensable for a dermatologist to make an accurate diagnosis. Additionally, a pathologic biopsy is a fundamental tool in this process.

Psoriasis vulgaris is an immune-mediated skin disease manifesting as scaly erythema or plaques. The histopathology of this condition shows an evenly thickened epidermis with neutrophil exocytosis and the formation of Munro microabscesses. The stratum corneum is thickened, hyperkeratotic, and contains neutrophils. Secukinumab is a biologic agent that is increasingly being utilized in clinical practice as an interleukin-17 inhibitor, as evidenced in numerous psoriasis treatment guidelines, which identify this agent as a primary therapeutic option for psoriasis [2]. While targeted therapies for psoriasis may prove efficacious in the treatment of several other immune system disorders, their potential benefit in the management of DLE remains a topic of contention [3]. In this report, we present a case of exacerbation of disseminated DLE due to misdiagnosis of psoriasis, exacerbated by treatment with secukinumab.

## Case presentation

**Figure 1:**
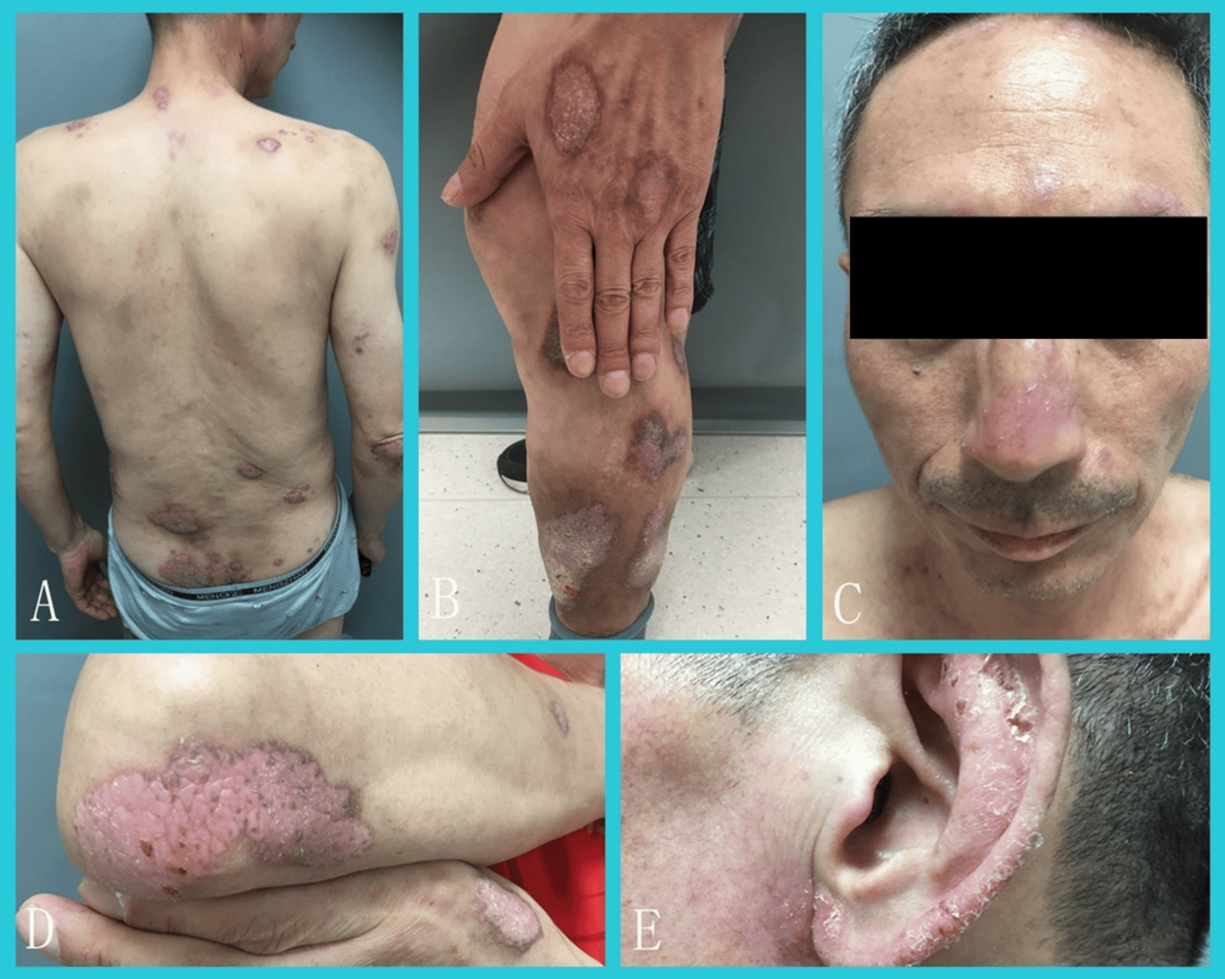
Clinical presentation. A-E: pale, reddish plaques with a few scales present on the face along with multiple purplish-red plaques on the extremities and back, with a rough surface and no visible ulceration or exudation.

A 52-year-old male patient presented with erythematous plaques causing itching on the face, trunk, and extremities persisting for over two years. He initially presented with itchy skin and cracked nails and gradually developed purple-red patches. He reported an increase in itching after exposure to sunlight. The patient was diagnosed with psoriasis at a tertiary care hospital and did not respond to topical mometasone ointment, tacrolimus ointment, or oral Chinese medicine. Over six months ago, the patient underwent a series of 20 subcutaneous injections of secukinumab 300 mg, which led to aggravated itching and no improvement in the lesions, despite the patient's apprehension towards treatment. Half a month ago, the patient switched to apremilast, which resulted in decreased itching but no reduction in erythema. The patient denied any previous history of tuberculosis, hepatitis, nephritis, or hereditary diseases within their family. Physical examination revealed no abnormalities. Dermatological examination revealed pale red patches with slight scaling on the face and numerous coarse-textured reddish-purple patches on the limbs and back, with no evidence of ulceration or exudation (Figure 1). The scalp showed no signs of alopecia or erythema, and the mucous membranes showed no signs of ulcers. The nails were dehiscent. Additionally, pale reddish plaques with minimal scaling were observed on both ears. Laboratory tests revealed a positive antinuclear antibody (ANA) titre of 1:320 and positive anti-Ro and anti-La antibodies. Anti-dsDNA, antihistone, and other anti-extractable nuclear antigen antibodies demonstrated negative results, and there was no depletion of complement C3 and C4 observed.

**Figure 2:**
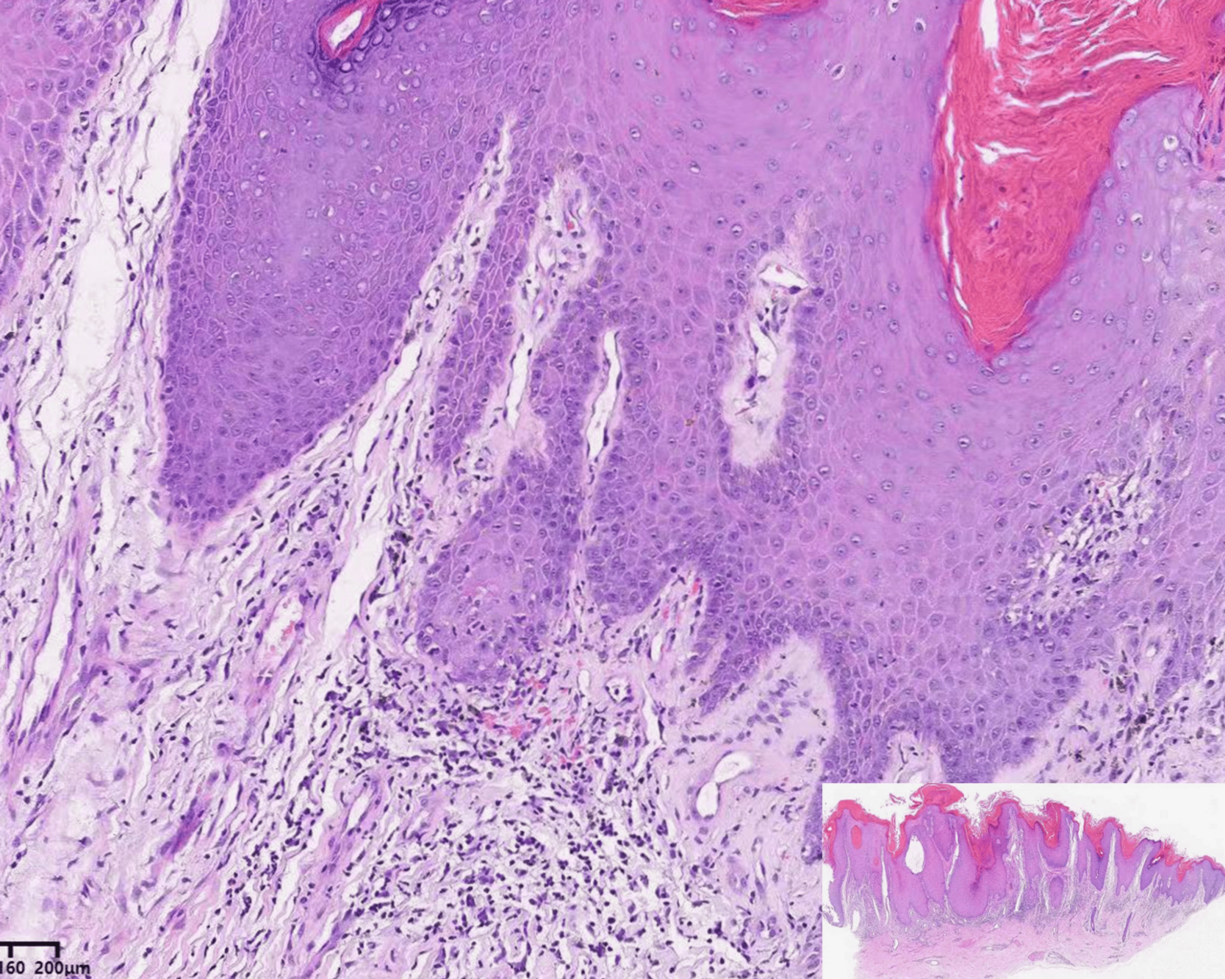
Hematoxylin and eosin staining pathology demonstration. The epidermis exhibits hyperkeratosis with foci of basal cell liquefaction and degeneration, accompanied by irregular epidermal hyperplasia, localized follicular keratinization, and follicular plugging. A perifollicular infiltrate and dense infiltration of lymphocytes and histiocytes and increased deposition of intercollagenous mucin accompany the basal liquefaction (H＆E, x200).

Pathological examination of lesions on the lower limbs revealed epidermal hyperkeratosis with focal basal cell liquefaction and degeneration, as well as perifollicular infiltrate, localized follicular keratinization, and follicular plugging. Additionally, basal liquefaction was observed, accompanied by dense lymphocyte and histiocyte infiltration and increased deposition of mucin between collagen. (Figure 2). These findings are consistent with the pathological diagnostic features of discoid lupus erythematosus.

The diagnosis of disseminated DLE was established through the examination of clinical symptoms, histopathological findings, and laboratory tests. The subject did not meet the diagnostic criteria for systemic lupus erythematosus. The patient was prescribed oral prednisone 30 mg per day and hydroxychloroquine 200 mg per day. After a month of treatment, the patient experienced a significant reduction in itching, and the red plaque was thinner than before. The patient is currently still under follow-up.

## Discussion

Disseminated DLE is an infrequent condition [4]. Its misdiagnosis as psoriasis is incidental but not surprising due to shared characteristics. Multiple erythematous areas, covered by similar scales, may increase the risk of misdiagnosis as psoriasis. DLE exhibits follicular keratosis beneath the scales, which causes dilation of the follicular orifices when the scales are removed. In contrast, psoriasis manifests as punctate hemorrhagic dots. Narrow-spectrum UV light is a typical treatment for psoriasis, but it can worsen DLE. In this case, direct application of biologics without this type of UV light led to erroneous outcomes. Diagnosis relies heavily on pathology, given that DLE symptoms comprise enlarged hair follicles and basal cell liquefaction, while psoriasis causes thinning of the granular layer and elongation and broadening of the epidermis [5].

The therapy of DLE with biologics is associated with limited experience. A review of the current literature showed that it comprises only two conditions: DLE along with psoriasis and drug-induced lupus with subsequent psoriasis in patients treated with biologics [6,7]. To the best of our knowledge, this is the inaugural instance of using secukinumab for the treatment of disseminated DLE due to misdiagnosis without contravening ethical principles.

Numerous paradoxical reactions have arisen during targeted therapy for psoriasis and atopic dermatitis, including the emergence of anti-nuclear antibodies (ANAs) and anti-dsDNA antibodies caused by TNF-α inhibitors in certain patients, resulting in autoimmunity. There are more reports of lupus-like reactions caused by TNF-α inhibitors compared to other biologics. It is hypothesized that the administration of TNF-α inhibitors may result in an unregulated increase in IFN-α, which in turn may induce lupus-like reactions [8]. These paradoxical reactions often demonstrate improvement following cessation of the drug or a switch to an alternate biologic [9]. This specific patient did not use adalimumab, but an aggravation of pruritus was experienced after sun exposure during the onset of the disease. Hence, it was not a paradoxical reaction caused by a biological agent.

Several biologic paradoxical reactions have been documented with interleukin (IL)-17 inhibitor therapy, comprising eczematous reactions, psoriasiform reactions, sarcoidosis-like reactions, vasculitis, and lupus-like disorders. The underlying pathomechanism in these complex diseases with the involvement of multiple immunological pathways is most likely a cytokine imbalance [10]. Several pro-inflammatory cytokines, such as TNF, IL-12, and IL-6, show increased levels in skin lesions of patients with cutaneous lupus erythematosus (CLE) compared to non-lesional skin or skin of healthy individuals [11]. Ustekinumab, an inhibitor of IL-12 and IL-23, may be effective in treating some patients with CLE. However, TNF inhibitors and IL-17 inhibitors can cause CLE-like lesions [12]. Secukinumab is a human monoclonal antibody that targets interleukin-17A. The potential cutaneous adverse reactions associated with its use have yet to be fully elucidated. After searching the Medline literature library, we found that secukinumab was associated with an increased risk of cutaneous lupus, as there were more adverse reports [6,7,13].

The rise in pro-inflammatory factors may be accountable for the unforeseen toxic effects associated with interleukin-17 inhibitors and may potentially culminate in the development of drug-induced lupus [7]. It is hypothesized that inhibition of IL-17A reduces differentiation of T helper type 17 cells and results in upregulation of IL-12 and IL-21 via an alternate pathway. Under conditions of IL-6 deficiency, the coexistence of TGF-β and IL-21 promotes CD4+ T cell differentiation into Th17 cells and IL-21 release. Two IL-6 receptor inhibitors, MRA003US and vobarilizumab, have been tested without success in treating SLE and/or CLE [3]. Nevertheless, the efficacy of interleukin-17 inhibitors in the treatment of CLE remains a controversial topic. It is conceivable that secukinumab may precipitate a progression of CLE, as do other pharmaceutical agents, by indirectly activating the innate immune system through the inhibition of autoantigen clearance [3].

## Conclusions

In conclusion, it is imperative that a comprehensive dermatological examination and pathological biopsy are conducted to ensure an accurate diagnosis prior to the utilization of biologics for the treatment of psoriasis. In this case, biopsy was crucial to identify disseminated DLE with psoriasis. While biologics have the potential to treat lupus, secukinumab may lead to worsening of DLE. Furthermore, interleukin-17 (IL-17) inhibitors may increase the risk of cutaneous lupus, and their efficacy in this regard must be evaluated in large samples.
